# Climate‐driven elevational variation in range sizes of vascular plants in the central Himalayas: A supporting case for Rapoport's rule

**DOI:** 10.1002/ece3.7744

**Published:** 2021-06-26

**Authors:** Jianchao Liang, Huijian Hu, Zhifeng Ding, Ganwen Lie, Zhixin Zhou, Paras Bikram Singh, Zhixiang Zhang, Shengnan Ji

**Affiliations:** ^1^ Laboratory of Systematic Evolution and Biogeography of Woody Plants School of Ecology and Nature Conservation Beijing Forestry University Beijing China; ^2^ Guangdong Key Laboratory of Animal Conservation and Resource Utilization Guangdong Public Laboratory of Wild Animal Conservation and Utilization Institute of Zoology Guangdong Academy of Sciences Guangzhou China; ^3^ Guangdong Eco‐Engineering Polytechnic Guangzhou China; ^4^ Biodiversity Conservation Society Nepal Lalitpur Nepal; ^5^ Museum of Beijing Forestry University Beijing China; ^6^ State Environmental Protection Key Laboratory of Regional Ecological Processes and Functions Assessment Chinese Research Academy of Environmental Sciences Beijing China

**Keywords:** climate variability, elevational gradient, Himalayas, Rapoport's rule, species range size, vascular plants

## Abstract

A fundamental yet controversial topic in biogeography is how and why species range sizes vary along spatial gradients. To advance our understanding of these questions and to provide insights into biological conservation, we assessed elevational variations in the range sizes of vascular plants with different life forms and biogeographical affinities and explored the main drivers underlying these variations in the longest valley in China's Himalayas, the Gyirong Valley. Elevational range sizes of vascular plants were documented in 96 sampling plots along an elevational gradient ranging from 1,800 to 5,400 m above sea level. We assessed the elevational variations in range size by averaging the range sizes of all recorded species within each sampling plot. We then related the range size to climate, disturbance, and the mid‐domain effect and explored the relative importance of these factors in explaining the range size variations using the Random Forest model. A total of 545 vascular plants were recorded in the sampling plots along the elevational gradient. Of these, 158, 387, 337, and 112 were woody, herbaceous, temperate, and tropical species, respectively. The range size of each group of vascular plants exhibited uniform increasing trends along the elevational gradient, which was consistent with the prediction of Rapoport's rule. Climate was the main driver of the increasing trends of vascular plant range sizes in the Gyirong Valley. The climate variability hypothesis and mean climate condition hypothesis could both explain the elevation–range size relationships. Our results reinforce the previous notion that Rapoport's rule applies to regions where the influence of climate is the most pronounced, and call for close attention to the impact of climate change to prevent species range contraction and even extinction due to global warming.

## Graphical abstract

Vascular plant range size increased along the elevational gradient in the central Himalayas as predicted by Rapoport's rule. Both climate variability and mean climate condition were the major determinants underlying the elevational variation in range size.

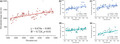

## 1 INTRODUCTION

Species range size is a fundamental unit in macroecology (Böhm et al., 2017). Understanding variation in species range size along spatial gradients is of primary importance in the study of climate change, biodiversity patterns, gene flow, and extinction mechanisms. A well‐known theory about spatial variation in species range size is Rapoport's rule, which states that species range size is positively correlated with latitude and elevation, that is, species at higher latitude or elevation have larger range size than those at lower latitude or elevation (Stevens, 1989). However, despite early evidence from the Northern Hemisphere (e.g., Arita et al., 2005; Blackburn & Gaston, 1996; Gaston et al., 1998; Letcher & Harvey, 1994), further studies from other regions yielded complex and partial support for this rule (e.g., Feng et al., 2016; Hawkins & Diniz‐Filho, 2006; Whitton et al., 2012), suggesting that this rule might be a regional phenomenon dependent on the local environment (Whitton et al., 2012). Therefore, recent attention has shifted from simply documenting variation in range size to exploring the drivers of variation.

Various studies have been conducted to understand the association between variation in range size and environmental factors such as climate (e.g., Sheldon & Tewksbury, 2014; Whitton et al., 2012), disturbance (e.g., Borkowski et al., 2016; Lozada et al., 2008), and the mid‐domain effect (MDE; e.g., Luo et al., 2011). Climate appears to be most important driver of both latitudinal and elevational variations in range size. Several hypotheses have been proposed to explain the climate–range size relationship, among which the climate variability hypothesis is the most commonly accepted (Pintor et al., 2015; Whitton et al., 2012). This hypothesis was first proposed by Stevens in 1989 and was believed to be the underlying mechanism of Rapoport's rule (Stevens, 1989, 1992). Stevens (1989) stated that climate, specifically temperature, is more variable at higher latitudes and elevations. Greater climatic variability favors species with wider tolerance and larger range size, thus leading to a positive relationship between range size and latitude and elevation. The mean climate condition hypothesis is another prominent explanation for the climate–range size relationships, which is supposed to cooperate with climate variability hypothesis to generate increasing trends of range size (Luo et al., 2011). The mean climate condition hypothesis proposes that species living at higher latitudes or elevations are not only subjected to greater climatic variation but also to lower mean climate conditions; thus, they tend to be geographically widely distributed (Jiang & Ma, 2014; Luo et al., 2011). In addition to contemporary climate, historical climate, such as the Quaternary climate, has also been proposed as an explanation for range size variations based on the premise that historical climate oscillations select for species with greater physiological tolerance and adaptability (Jansson, 2003; Araújo et al., 2008).

Apart from climatic factors, disturbance and MDE are also believed to influence species range size. The disturbance hypothesis proposes that anthropogenic threats might lead to population declines and extinctions, thus constraining species range size (Whitton et al., 2012). The MDE postulates that the range of species who live near the edge of the domain will be truncated by the domain boundaries, leading to smaller mean range size near the boundaries and larger mean range size at the domain center. Therefore, it predicts a mid‐peak pattern in species range size, regardless of the ecological factors (Feng et al., 2016; Luo et al., 2011; Sandel & McKone, 2006).

In addition to environmental factors, variations in species range size might also be associated with life form and biogeographical affinities, as these reflect species ecophysiological traits and evolutionary history, and may thus affect their response to environmental variation. For example, compared with herbaceous plants, woody plants tend to have a lower adaptability due to their longer reproductive cycles and slower accumulation rate of genetic changes (Smith & Beaulieu, 2009) and might thus be more sensitive to environmental gradients. Similarly, tropical taxa, which have experienced a more stable climatic environment in their evolutionary history, may be more susceptible to climatic variation and thus be more supportive of increasing trend in species range size with latitude and elevation (Feng et al., 2016; McCain, 2009). However, little research has been conducted to examine the variations in species range size in terms of the influence of life form and biogeographical affinities (but see Feng et al., 2016; Zhou et al., 2019).

As one of the world's 34 biodiversity hotspots, the Himalayas encompass a diverse range of eco‐climatic zones, and have been the focus of various ecological and biogeographical studies. Particularly in the central Himalayas, where the towering mountains block the incoming moisture from the Indian Ocean, a series of north–south valleys present rich biodiversity and conspicuous elevational environmental gradients at a small spatial scale. This makes them an ideal place for studying the underlying mechanisms of spatial variation in species range size and examining the validity of Rapoport's rule. However, while numerous studies have explored the elevational variation in species richness and its drivers in the Himalayas (e.g., Acharya et al., 2011; Kluge et al., 2017; Manish et al., 2017; Sun et al., 2020; Yang et al., 2018), corresponding studies on species range size are limited. As understanding range size variations is a prelude to effective biodiversity conservation (Luo et al., 2011), bridging this research gap will not only help address the theoretical issue, but also contribute to conservation practices in this high‐profile region.

Vascular plants have long been considered an excellent subject for studying spatial variations in range size because of their wide distribution and ease of observation. In this study, we aimed to examine the elevational variations in range sizes of vascular plants with different life forms and biogeographical affinities, and to explore the role of climate, disturbance, and MDE on such variations, based on a detailed field survey in the Gyirong Valley, which is the longest valley in China's central Himalayas. As species range size is considered to be closely associated with species richness (Stevens, 1992), and as climate has been reported as the primary determinant of species richness in the Himalayas (Bhattarai & Vetaas, 2003; Liang et al., 2020; Manish et al., 2017; Sun et al., 2020), we hypothesized that climatic factors would better explain the elevational variation in range sizes of vascular plants in the Gyirong Valley than other factors. In this case, considering that Rapoport's rule is supported in regions with the most pronounced influence of climate (Pintor et al., 2015), we further speculated that the range size of vascular plants would increase with elevation as the rule predicts, particularly for woody and tropical species, which are more sensitive to climatic variation.

## 2 METHODS

### 2.1 Study area and field sampling

**FIGURE 1:**
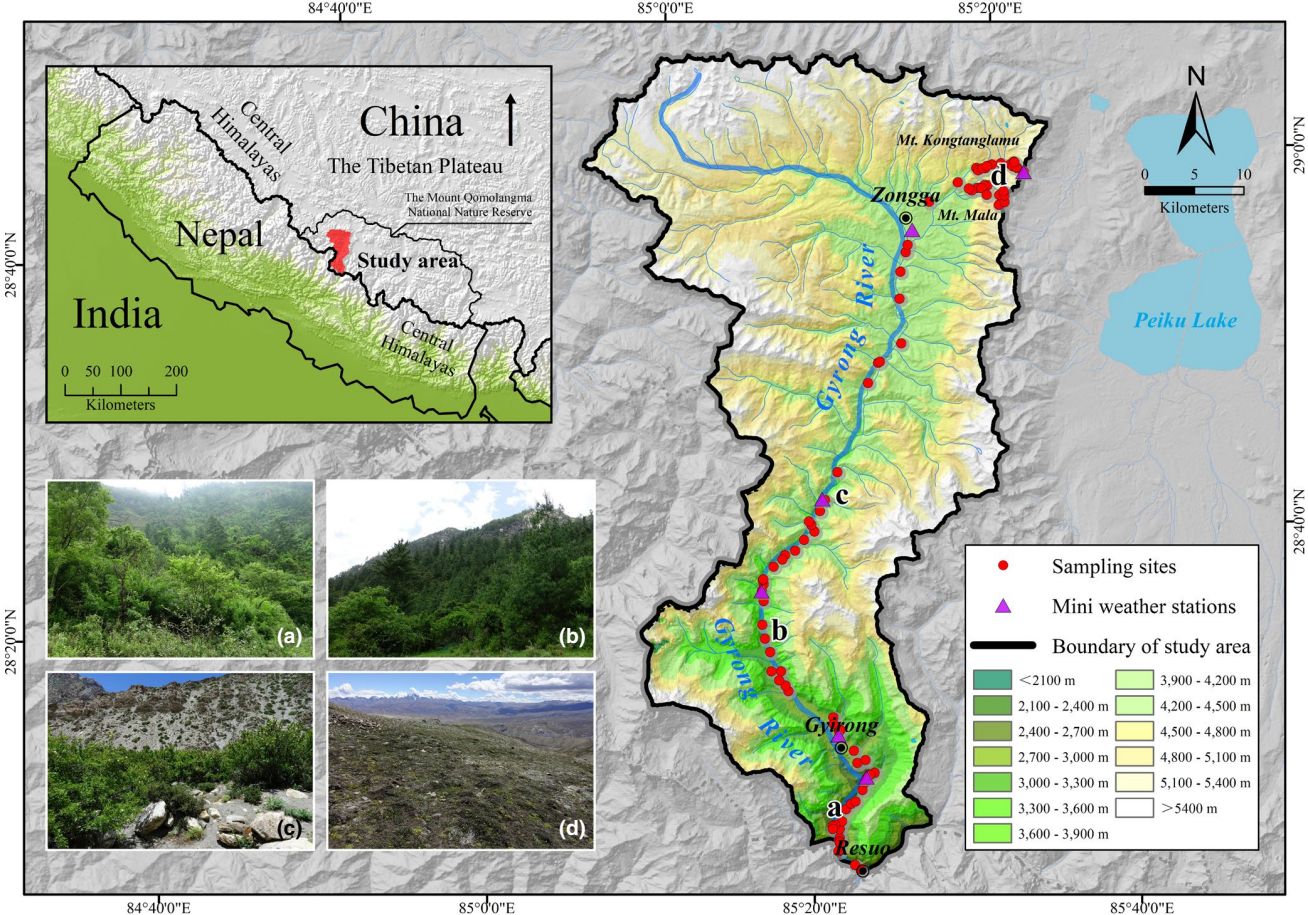
Map of the study area showing the locations of the 96 sampling plots and 6 mini weather stations along the Gyirong Valley. The letters correspond to the vegetation zones shown in the lower left corner of the map: (a) evergreen broadleaf forest; (b) subalpine coniferous forest; (c) alpine bush and coryphilum; (d) alpine tundra with sparse herbs

The Gyirong Valley (28°16′‐29°00′N, 84°56′‐85°24′E) is located in the southern part of the Tibetan Plateau in China, bordering with the northern part of Nepal (Figure 1). The valley extends over an area of 90 km and spans an elevational range of 1,840 to 7,341 m above sea level (a.s.l.). Due to the influence of the Indian Ocean monsoon, the valley exhibits steep environmental gradients and distinct elevational vegetation zones, which can be divided into evergreen broadleaf forests (1,800–2,500 m a.s.l.), coniferous and broadleaf mixed forests (2,500–3,300 m a.s.l.), subalpine coniferous forests (3,300–3,900 m a.s.l.), alpine bush and coryphilum (3,900–4,700 m a.s.l.), alpine tundra with sparse herbs (4,700–5,400 m a.s.l.), and a scree and nival zone above 5,400 m a.s.l.

Our study was conducted along an elevation gradient, from Resuo village at 1,800 m a.s.l. to Mt. Kongtanglamu and Mt. Mala at 5,400 m a.s.l. Elevations lower than 1,800 m a.s.l. and higher than 5,400 m a.s.l. were excluded from the study due to geopolitical restrictions and the scree and nival zone, where very few creatures can survive. Field surveys were carried out in July and August 2018 using 96 sampling plots along this elevation gradient. The sampling plots were established based on the most common physiognomic vegetation and topographic accessibility. In each plot, the vascular plants were exhaustively inventoried (for 2–4 hr by 5 individuals) within a quadrat of 400 m^2^, following Fang et al. (2009). Species that could not be identified in the field were taken to the Museum of Beijing Forestry University for identification.

### 2.2 Species grouping

Life form of each species was divided into woody species (i.e., trees and shrubs) and herbaceous species (i.e., herbs and climbers) based on the field survey and species description on monographs (e.g., Flora of China, www.efloras.org; Flora of Pan‐Himalayas, www.flph.org). Following Feng et al. (2016), biogeographical affinity of each species was classified as temperate species, tropical species, and cosmopolitan species, based on a classification system of biogeographical affinities proposed by Wu (1991). Species with distribution centers in northern temperate regions (i.e., East and North Asia, America, old world temperate regions, temperate Asia, Mediterranean, west to central Asia, Central Asia, and East Asia) were considered as temperate species, while species with distribution centers in pantropic regions (i.e., tropical Asia and tropical America, old world tropic regions, tropical Asia to tropical Australia, tropical Asia to tropical Africa, and tropical Asia) were considered as tropical species. Species that span from tropics to temperate regions and have no obvious distribution centers were considered as cosmopolitan species. Only temperate and tropical species were considered when assess the influence of biogeographical affinities on range size variation (Feng et al., 2016).

### 2.3 Species range size

For each species, the range size was estimated as the difference between the maximum and minimum elevation of the sampling plot where it was recorded. Following Steven's method (Stevens, 1992), we averaged the range size for each group of vascular plants within each sampling plot for the subsequent analyses.

### 2.4 Environmental variables

Eight environmental variables were used to examine the effect of mean climate condition, climate variability, historical climate change, disturbance, and MDE on the elevational variation in vascular plant range sizes.

The mean climate condition variables included mean annual temperature (MAT) and mean annual precipitation (MAP). The climate variability variables included temperature seasonality (TS) and mean annual temperature range (MATR). MAT, MAP, TS, and MATR were obtained from six mini weather stations established along the Gyirong Valley, from 2016 to 2018 (at 2,457, 2,792, 3,368, 3,740, 4,140, and 5,230 m a.s.l.; Figure 1). We averaged the 3‐year data of the four variables for each station and extrapolated this data for the entire study area using Kriging interpolation in a GIS environment (Hu et al., 2018). For each of the four variables, we used the grid value corresponding to the location of the 96 sampling plots.

The variables of historical climate change included changes in mean annual temperature (TC) and precipitation (PC) between the present and the Last Glacial Maximum (LGM; approximately 22,000 years ago). The annual temperature and precipitation of the LGM were derived from the average of three global climate models (GCMs), namely CCSM4, MIROC‐ESM, and MPI‐ESM‐P, which were obtained from the WorldClim dataset (www.worldclim.org).

The disturbance was quantified using the inverse distance weighted interpolation of human population (POP), based on the assumption that the larger the population, the greater its disturbance to surrounding environment, and such disturbance decreases with increasing distance. The POP data were derived from the demographics of villages and towns in the Gyirong Valley, which were provided by the authority of the Mount Qomolangma National Nature Reserve. We extracted POP from the interpolation for each of the sampling plots.

The MDE was tested using the predicted mean range size under boundary constraints, which was calculated by reshuffling the species ranges within the elevational gradient (1,800–5,400 m a.s.l.). The calculation was performed using the Monte Carlo simulation and implemented in Range Model 5 software (Colwell, 2008). We ran 1,000 Monte Carlo simulations of empirical range sizes sampled without replacement to ensure that all species were reshuffled, and used average of the simulations as the predicted mean range size (Luo et al., 2011).

### 2.5 Statistical analysis

Linear regressions were calculated to assess the relationship between the elevation and average species range size of sampling plots. Rapoport's rule was considered to be supported for regressions with a positive relationship (Moreno et al., 2010).

Relationships between species range size and each environmental variable were assessed using ordinary least squares (OLS) models. The simultaneous autoregressive (SAR) model was further used to account for spatial autocorrelation in variables. All variables were standardized (mean = 0 and standard deviation = 1) to yield comparable regression coefficients for OLS and SAR models.

The Random Forest model was used to explore the relative importance of each environmental variable in explaining the elevational variations in species range size. We selected this model as it does not require any assumptions in the data (e.g., normality in errors and homoscedasticity) and can better manage multicollinearity and nonlinear relationships among variables, unlike most traditional methods, such as GLMs (Breiman, 2001; Feng et al., 2017). We ran the Random Forests model 1,000 times and assessed the relative importance of each environmental variable using the average of the percentage increase in mean squared error (%IncMSE) of the models. The %IncMSE was calculated by repeated permutation of each environmental variable, which represents the increase in prediction error caused by each individual variable.

These above analyses were performed in the R environment, using “vegan,” “spdep,” and “randomForest” packages.

## 3 RESULTS

### 3.1 General description

A total of 545 vascular plants belonging to 106 families and 339 genera were recorded from 96 sampling plots along the elevational gradient in the Gyirong Valley. Of these, 158 were woody (28.99%) and 387 were herbaceous (71.01%), whereas 337 were temperate (61.83%) and 112 were tropical (20.55%) species.

**FIGURE 2:**
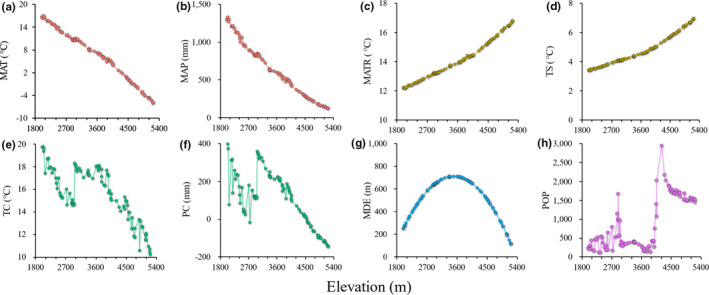
Elevational variations in (a) MAT, mean annual temperature; (b) MAP, mean annual precipitation; (c) MATR, mean annual temperature range; (d) TS, temperature seasonality; (e) TC, temperature change between the present and the Last Glacial Maximum; (f) PC, precipitation change between the present and the Last Glacial Maximum; (g) MDE, mid‐domain effect; (h) POP, human population

MAT and MAP decreased sharply with increasing elevation, whereas MATR and TS increased monotonically. TC and PC exhibited a similar pattern, presenting a general decrease with an intermediate trough at 2,700 m a.s.l. POP exhibited a bimodal pattern, with peaks at 2,700 and 4,200 m a.s.l. corresponding to the towns of Gyirong and Zongga, respectively. The MDE of all groups of vascular plants exhibited a mid‐peak pattern (Figure 2, Figure S1).

### 3.2 Elevational trends of species range size

**FIGURE 3:**
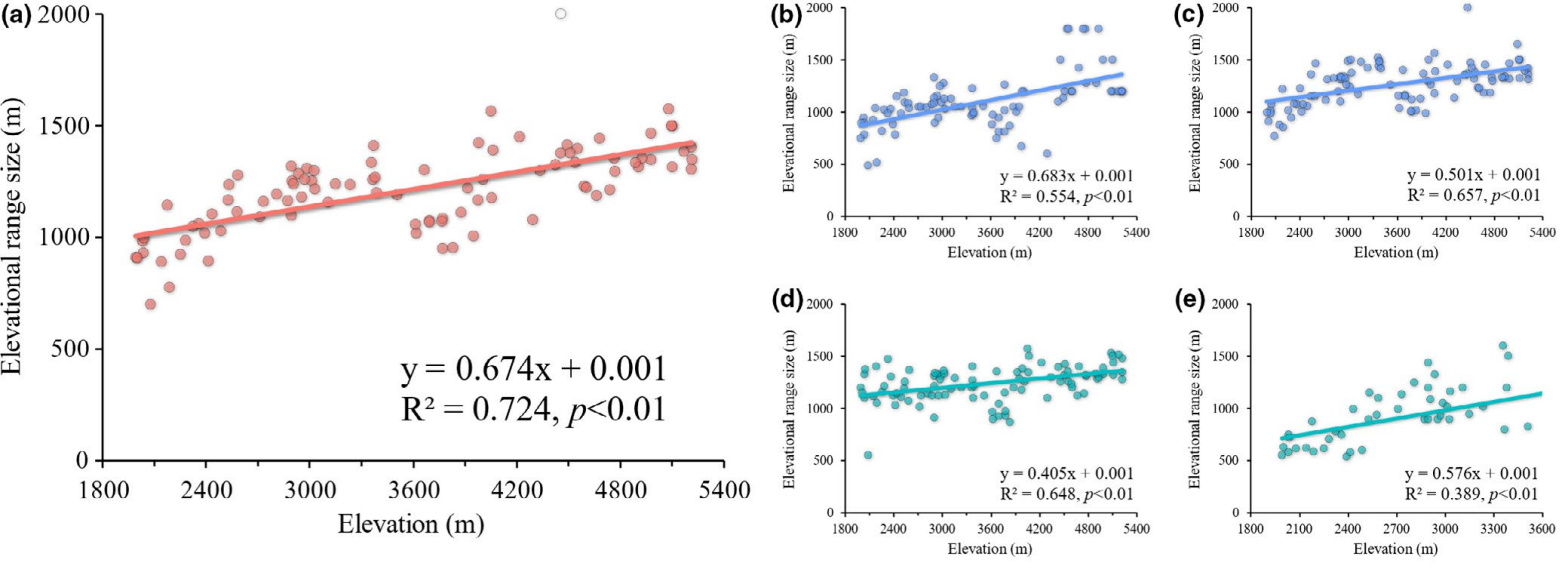
Elevational trends in the range size of (a) overall species, (b) woody species, (c) herbaceous species, (d) temperate species, and (e) tropical species in the Gyirong Valley

The species range size was positively correlated with elevation for all groups of vascular plants and presented uniform increasing trends along the elevational gradient as predicted by Rapoport's rule (Figure 3). Woody and tropical species were found to have a relatively stronger range size–elevation relationship with a higher regression coefficient.

### 3.3 Relationships between species range size and environmental variables

The OLS and SAR models yielded similar results regarding the relationship between species range size and environmental variables, although the correlation decreased when spatial autocorrelation was taken into account (Tables 1 and 2). For all groups of vascular plants, almost all environmental variables were significantly correlated with species range size along the elevational gradient, except for MDE and POP. Among these, MAT, MAP, TC, and PC presented negative relationships with species range size, whereas MATR and TS exhibited a positive relationship.

**TABLE 1:** The ordinary least squares (OLS) models for each environmental variable and species range size of all groups of vascular plants

	Overall species			Woody species			Herbaceous species			Temperate species			Tropical species		
	Coef	*SE*	Adj *R* ^2^	Coef	*SE*	Adj *R* ^2^	Coef	*SE*	Adj *R* ^2^	Coef	*SE*	Adj *R* ^2^	Coef	*SE*	Adj *R* ^2^
MAT	−0.734***	0.079	0.601	−0.724***	0.089	0.605	−0.705***	0.089	0.584	−0.675***	0.096	0.559	−0.665***	0.099	0.589
MAP	−0.720***	0.080	0.585	−0.719***	0.091	0.591	−0.681***	0.087	0.569	−0.670***	0.097	0.551	−0.673***	0.098	0.591
MATR	0.770***	0.078	0.619	0.696***	0.090	0.576	0.733***	0.091	0.598	0.702***	0.096	0.579	0.682***	0.101	0.570
TS	0.789***	0.076	0.630	0.700***	0.089	0.574	0.767***	0.091	0.617	0.686***	0.095	0.562	0.688***	0.100	0.576
TC	−0.539***	0.085	0.482	−0.489***	0.090	0.437	−0.513***	0.094	0.463	−0.465**	0.099	0.418	−0.485***	0.134	0.436
PC	−0.487***	0.090	0.430	−0.557***	0.095	0.490	−0.565***	0.098	0.498	−0.501*	0.101	0.452	−0.491***	0.126	0.442
MDE	0.376	0.105	0.371	0.384	0.093	0.388	0.375	0.094	0.366	0.285	0.102	0.301	0.288	0.102	0.314
POP	0.298	0.085	0.320	0.284	0.093	0.309	0.293	0.095	0.324	0.334	0.095	0.341	0.303	0.099	0.317

Note

The strength of correlation was measured by regression coefficient (Coef).

Abbreviations: Adj *R* ^2^, adjusted *R* ^2^; MAP, mean annual precipitation; MAT, mean annual temperature; MATR, mean annual temperature range; MDE, mid‐domain effect; PC, change in mean annual precipitation between the present and the Last Glacial Maximum; POP, human population; *SE*, standard error; TC, change in mean annual temperature between the present and the Last Glacial Maximum; TS, temperature seasonality.

* *p* < .05

** *p* < .01

*** *p* < .001.

**TABLE 2:** The simultaneous autoregressive (SAR) models for each environmental variable and species range size of all groups of vascular plants

	Overall species			Woody species			Herbaceous species			Temperate species			Tropical species		
	Coef	*SE*	AIC	Coef	*SE*	AIC	Coef	*SE*	AIC	Coef	*SE*	AIC	Coef	*SE*	AIC
MAT	−0.627***	0.091	202.20	−0.613***	0.102	204.79	−0.597***	0.103	222.51	−0.552***	0.121	252.91	−0.558***	0.134	230.42
MAP	−0.604***	0.088	202.18	−0.609***	0.101	205.70	−0.584***	0.101	221.92	−0.569***	0.115	254.45	−0.562***	0.128	227.17
MATR	0.662***	0.093	203.46	0.583***	0.104	204.24	0.629***	0.105	223.13	0.598***	0.123	252.88	0.578***	0.102	222.42
TS	0.686***	0.094	202.06	0.596***	0.105	202.89	0.659***	0.107	222.36	0.578***	0.124	251.83	0.587***	0.103	221.68
TC	−0.476***	0.117	223.19	−0.446***	0.115	222.16	−0.457***	0.127	243.45	−0.420***	0.152	260.33	−0.440***	0.114	206.11
PC	−0.439***	0.144	235.65	−0.509***	0.154	216.9	−0.512***	0.150	234.00	−0.455***	0.115	263.05	−0.408***	0.083	217.76
MDE	0.339***	0.185	251.72	0.334***	0.195	240.79	0.322***	0.093	249.54	0.230***	0.162	282.64	0.213***	0.116	287.36
POP	0.293***	0.089	252.56	0.286***	0.112	243.25	0.279***	0.098	257.70	0.324***	0.113	251.39	0.296***	0.134	250.13

Note

The strength of correlation was measured by regression coefficient (Coef).

Abbreviations: AIC, Akaike information criterion; MAP, mean annual precipitation; MAT, mean annual temperature; MATR, mean annual temperature range; MDE, mid‐domain effect; PC, change in mean annual precipitation between the present and the Last Glacial Maximum; POP, human population; *SE*, standard error; TC, change in mean annual temperature between the present and the Last Glacial Maximum; TS, temperature seasonality.

* *p* < .05

** *p* < .01

*** *p* < .001.

### 3.4 Relative importance of each environmental variable

**FIGURE 4:**
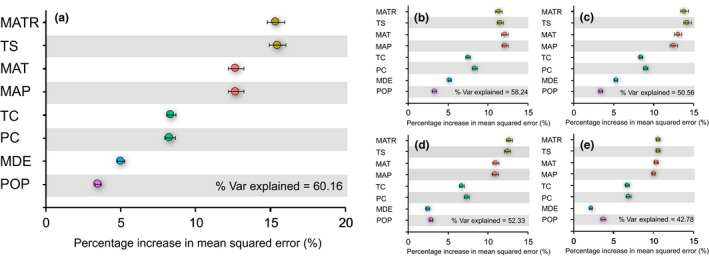
The average percentage increase in mean squared error of each environmental variable in 1,000 Random Forest models for (a) overall species, (b) woody species, (c) herbaceous species, (d) temperate species, and (e) tropical species

The Random Forest model explained 60.16%, 58.24%, 50.56%, 52.33%, and 42.78% of the variation in range size across all, woody, herbaceous, temperate, and tropical species, respectively. In general, MATR, TS, MAT, and MAP were the most important variables for explaining the elevational variation in range size across all groups of vascular plants (Figure 4). TC and PC also played supplementary roles in determining the range size of vascular plants, whereas MDE and POP only weakly explained the variation in range size.

## 4 DISCUSSION

The range size of all groups of vascular plants exhibited uniform increasing trends along the elevational gradient of the Gyirong Valley. As predicted, climatic factors played a greater role in shaping these trends than did other factors. Both the climate variability hypothesis and mean climate condition hypothesis could explain the elevation–range size relationships. Therefore, as expected, Rapoport's rule was supported regardless of the life form and biogeographical affinities.

### 4.1 The influence of life form and biogeographical affinities

Life form and biogeographical affinities are known to affect species response to environmental gradients; however, studies on how they influence the elevational variation in species range size are relatively scarce (but see Feng et al., 2016; Zhou et al., 2019). In Mount Kenya, Zhou et al. (2018) observed a monotonically increasing trend for range size of herbaceous species, but a distinct right‐skewed unimodal trend for range size of woody species. In Nepal, Feng et al. (2016) reported that the tropical species partially supported Rapoport's rule, whereas the temperate species did not support it. However, in the Gyirong Valley of the central Himalayas, the range sizes of vascular plants across different life forms and biogeographical affinities exhibited uniform increasing trends, although woody and tropical species presented a relatively stronger range size–elevation relationship as they are more sensitive to environmental gradients. Zhou et al. (2018) attributed the decrease in the range size of woody species at higher elevations of Mount Kenya to a greater proportion of endemic species. However, in the Gyirong Valley, both richness and proportion of endemic species exhibited a left‐skewed unimodal pattern (Figure S2). Higher elevation was characterized by widely distributed nonendemic species, such as *Spiraea alpina*, *Potentilla parvifolia*, and *Lonicera spinosa*, which might account for the aforementioned difference in the elevational trends of the range size of woody species. On the other hand, it must be noted that none of the climatic variability variables in Nepal showed an increasing trend with elevation (Feng et al., 2016), whereas all climatic variability variables in the Gyirong Valley increased monotonically along the elevational gradient. Given that the increasing climatic variability gradient is indispensable for Rapoport's rule, this rule was equivocally supported in Feng's study but was strongly supported in our study. Collectively, our results suggest that the influence of life form and biogeographical affinities on range size variation might be environment‐dependent.

### 4.2 The role of different environmental factors

Climate, particularly contemporary climate, played a greater role in shaping the increasing trends of vascular plant range sizes in the Gyirong Valley than other environmental factors. This result echoes the predominance of climate in determining the elevational gradient of plant richness in the Himalayas (Bhattarai & Vetaas, 2003; Liang et al., 2020; Manish et al., 2017; Sun et al., 2020). This could be attributed to the fact that the Himalayas have a more distinct and complete vertical climatic gradient compared with most other mountains at the same latitude because of its unparalleled elevational range. For example, all contemporary climatic variables, including MAT, MAP, TS, and MATR, presented monotonic trends along the elevational gradient in the Gyirong Valley. TS and MATR were the most important variables influencing the range size of all groups of vascular plants and exhibited a significant positive relationship with range size, which provides supporting evidence for the climate variability hypothesis. In addition, the mean climate condition hypothesis was also supported as MAT and MAP were important variables exhibiting a significant negative relationship with range size. Our results suggesting that the impacts of climate variability and mean climate conditions on range size variation are inseparable. With increasing elevation in the Gyirong Valley, species were subjected to both increasing climate variability and declining climate conditions and thus tend to have greater adaptability and larger range.

Apart from the primary importance of contemporary climate, historical climate plays supplementary roles in shaping the elevational trends of range size. To our surprise, species range size shows negative relationship with TC and PC. It is possible that historical climate oscillations could have promoted speciation (Hewitt, 1996, 2004; Leprieur et al., 2011; Zhao et al., 2016), resulting in a higher proportion of narrowly distributed endemic species at lower elevations in the Gyirong Valley.

Disturbance factors and MDE contributed minimally to the elevational variation in range size. As the Gyirong Valley is located within the Mount Qomolangma National Nature Reserve (Figure 1), human activities are restricted in the vicinity of Gyirong and Zongga towns; thus, they have a lesser impact on the elevational range size of vascular plants. The influence of MDE was affected by the range size of species, with large‐range species being more sensitive to MDE (Colwell et al., 2004). In the Gyirong Valley, over 90% of the vascular plants have a small range of less than 1,800 m (half of the sampling gradient), which could explain the weak explanatory power of MDE.

### 4.3 The applicability of Rapoport's rule

Since its formulation, the validity of Rapoport's rule has been controversial. The applicability of this rule varies greatly in different regions of the world. In general, the rule appears to be more well defined in the Northern Hemisphere and at higher latitudes than in the Southern Hemisphere and at lower latitudes (Böhm et al., 2017). It must be noted that when Stevens first introduced Rapoport's rule in 1989, he emphasized that the rule should apply to species inhabiting regions with conspicuous gradients of climate variability (Stevens, 1989). Further studies also confirmed the necessity for climate variability to the validity of Rapoport's rule. For example, Whitton et al. (2012) suggested that the primary importance of climate variability may explain why Rapoport's rule is largely restricted to northern latitudes, as this is where temperature seasonality is the most pronounced. Similarly, Pintor et al. (2015) attributed the absence of Rapoport's rule in Australia to the complex climate pattern across the entire continent, with minimum and maximum temperatures varying considerably at any given latitude. In our study, climate variability exhibited a monotonically increasing pattern along the elevational gradient in the Gyirong Valley and was the most influential factor affecting the elevational variation in range size of all groups of vascular plants. Therefore, it is not surprising that Rapoport's rule is supported regardless of the life form and biogeographical affinities. We believe the influence of life form and biogeographical affinities on the applicability of Rapoport's rule might be environment‐dependent, and confirm the previous findings that climate variability is the ultimate determinant of the validity of this rule.

### 4.4 Conservation implication

As climate plays a significant role in determining species range, there is an urgent need to focus on the impact of climate change. It has been widely reported that climate change will force species to shift their range upward along the mountains (Feeley & Silman, 2010; Feeley et al., 2011; Rehm, 2014), leading to a shift in elevational biodiversity hotspots (Wu et al., 2016). On the other hand, climate change has been implicated in species range contractions on several mountains. For example, Engler et al. (2011) assessed the impacts of climate change on 2,632 plant species across all major European mountain ranges and predicted that 36%–55% of alpine species, 31%–51% of subalpine species, and 19%–46% of montane species will lose more than 80% of their suitable habitat by 2070–2100. As the Himalayas are among the most sensitive regions to climate change (Xu et al., 2009), our concerns regarding the susceptibility and adaptation of plants to climate change are warranted. Specifically, given the extreme environmental conditions and geographic constraints at the high elevation of the Gyirong Valley, plants are likely to fail in expanding their upper limit, while their lower range limit increases with their upward range shift under climate change, leading to range contraction and even extinction of narrow‐range species. Considering that the response to climate change is species‐specific, long‐term monitoring is imperative for understanding the impact of climate change on local biodiversity.

## 5 CONCLUSIONS

In the Gyirong Valley of the central Himalayas, the range size of vascular plants across different life forms and biogeographical affinities was found to increase uniformly along the elevational gradient, which was consistent with the prediction of Rapoport's rule. Climate, particularly contemporary climate, was the main driver of the increasing trends of vascular plant range sizes. Both the climate variability hypothesis and mean climate condition hypothesis could explain the elevational variation in range size. Our results reinforce the previous notion that Rapoport's rule applies to regions where the influence of climate is the most pronounced. Such climate‐driven variations in range size call for close attention to the impact of climate change, which has been implicated in range contractions and even extinction of several taxa.

## CONFLICT OF INTEREST

None declared.

## AUTHOR CONTRIBUTIONS

**Jianchao Liang:** Conceptualization (equal); data curation (lead); formal analysis (lead); investigation (lead); methodology (lead); resources (lead); software (lead); validation (equal); visualization (lead); writing‐original draft (lead). **Huijian Hu:** Conceptualization (equal); funding acquisition (lead); supervision (equal). **Zhifeng Ding:** Formal analysis (supporting); funding acquisition (supporting); software (supporting); validation (supporting); writing‐original draft (supporting). **Ganwen Lie:** Formal analysis (supporting); investigation (supporting); visualization (supporting). **Zhixin Zhou:** Formal analysis (supporting); funding acquisition (supporting); software (supporting). **Paras Bikram Singh:** Writing–original draft (supporting). **Zhixiang Zhang:** Conceptualization (equal); funding acquisition (supporting); supervision (equal). **Shengnan Ji:** Conceptualization (supporting); funding acquisition (lead); supervision (supporting).

## Supporting information

Fig S1‐S2

## Data Availability

Data are available via the Dryad Digital Repository: https://doi.org/10.5061/dryad.ghx3ffbp0.
