# Supplementary material for: Climate‐driven elevational variation in range sizes of vascular plants in the central Himalayas: A supporting case for Rapoport's rule
Source: Ecol Evol. 2021 Jun 26;11(14):9385–95. doi: 10.1002/ece3.7744 (PMC8293715; doi:10.1002/ece3.7744)
Supplement: Supplementary file 1 — Fig S1‐S2 [file ECE3-11-9385-s001.docx]

**Supporting Information**

**
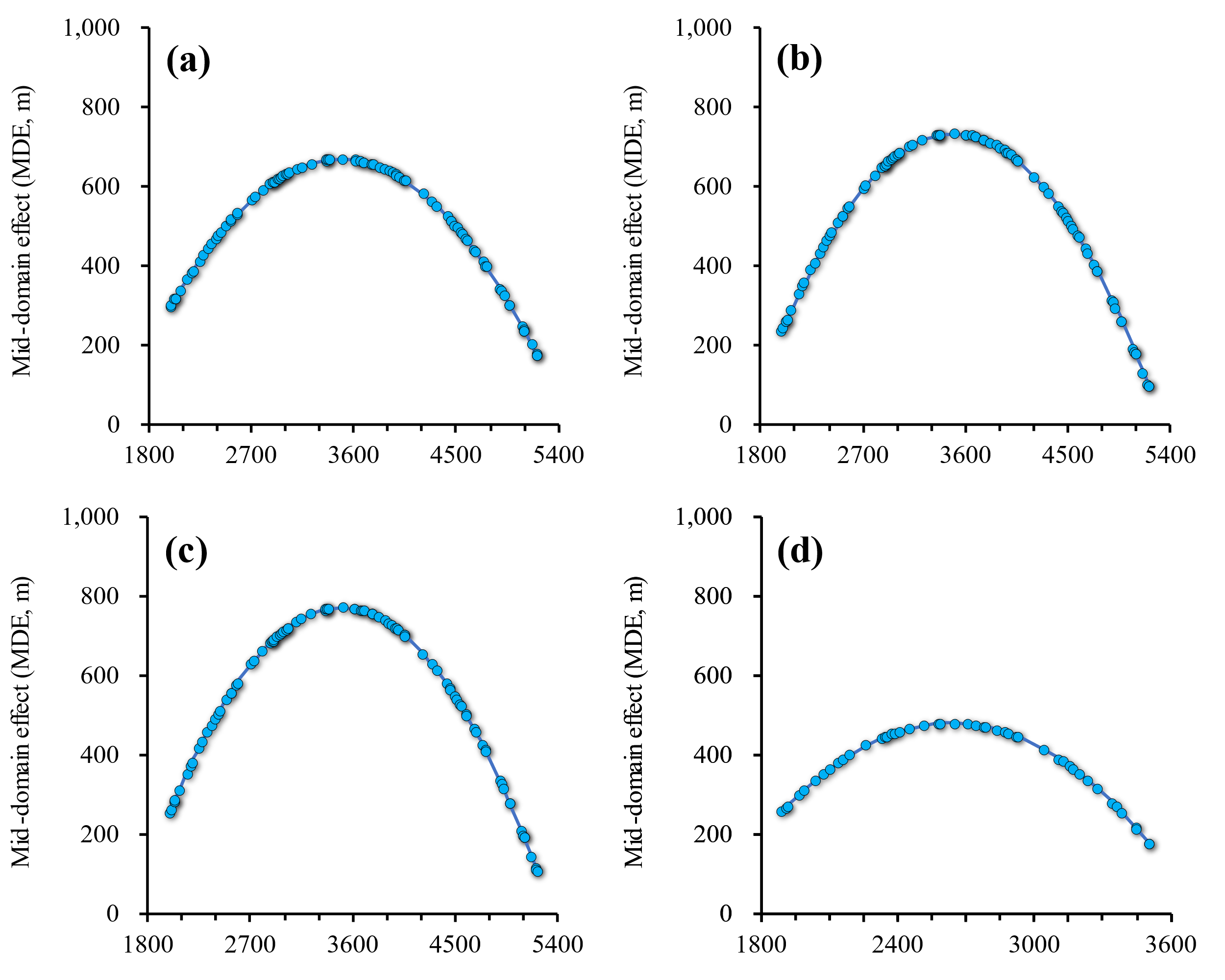
**

**Fig. S1** Simulated average range size under geometric constraints for (a) woody species, (b) herbaceous species, (c) temperate species, (d) tropical species.

**Fig. S2** The richness and percentage of endemic species along the elevational gradients of the Gyirong Valley.
